# High Oxygen Treatments Enhance the Contents of Phenolic Compound and Ganoderic Acid, and the Antioxidant and DNA Damage Protective Activities of *Ganoderma lingzhi* Fruiting Body

**DOI:** 10.3389/fmicb.2019.02363

**Published:** 2019-10-18

**Authors:** Qingying Dong, Yueyue Li, Gaoqiang Liu, Zhiwei Zhang, Huabin Zhou, Hailong Yang

**Affiliations:** ^1^School of Life and Environmental Sciences, Wenzhou University, Wenzhou, China; ^2^National Engineering Laboratory for Rice and By-Product Further Processing, Central South University of Forestry and Technology, Changsha, China

**Keywords:** *Ganoderma lingzhi*, high oxygen, ganoderic acid, free radical scavenging, DNA damage protection

## Abstract

*Ganoderma lingzhi* is a famous medicinal mushroom used as Chinese medicine or functional food and has been accepted across the globe. It is important to enhance the contents of bioactive compounds, which in turn improves the quality and biological activity of *G. lingzhi* fruiting body. In this work, freshly harvested *G. lingzhi* fruiting bodies were treated continuously with air or with 60 and 80% oxygen for 6 days. Samples were collected and determined initially and at 1 day interval during treatment. A high total ganoderic acid content of 29.44 g kg^–1^ was obtained in samples treated with 60% oxygen at day 3. Quantitative reverse transcriptase (qRT)-PCR and high-performance liquid chromatography (HPLC) analysis showed that the expression levels of hydroxymethylglutaryl-CoA synthase, squalene synthase, and oxidosqualene cyclase genes were substantially increased, resulting in the increase of ganoderic acids A, B, and C2 and ganoderenic acid B. The scavenging activities with 1,1-diphenyl-2-picrylhydrazyl radical, 2,2′-azino-bis(3-ethylbenzothiazoline-6-sulfonic acid) radical, hydroxyl radical, and superoxide radical and the DNA damage protective activity were also enhanced by high oxygen treatment. The results of this work provided a potential method to enhance the active metabolite synthesis in the fruiting body of *G. lingzhi*.

## Introduction

*Ganoderma lingzhi*, formerly named *Ganoderma lucidum*, has been used to strengthen health and treat disease in traditional Chinese and Japanese medicine for more than 2,000 years (Bishop et al., 2015; Si et al., 2019). Ganoderic acids, a class of high oxidized lanostane-tetracyclic triterpenes, are deemed to be one of the most important bioactive secondary metabolites of *G. lingzhi*. Since ganoderic acids A and B were first reported by Kubota et al. (1982), a series of ganoderic acids have been isolated and reported (Xu et al., 2010b), and most of them exhibit significant pharmacological properties such as anti-HIV-1, antimicrobial, antioxidant, antitumor, hepatoprotection, hypoglycemic, and neurotrophic activities (Cör et al., 2018). However, low yields are limiting the wide application of ganoderic acids as a medicine to treat diseases. To improve the biosynthesis of ganoderic acids, several bioprocessing strategies such as media optimization (Xu et al., 2008), shaking combining static culture (Xu et al., 2010a), and overexpression of squalene synthase gene (Zhou et al., 2014) have been developed. These techniques are all based on mycelia growth and submerged culture of *G. lingzhi*. However, about 80–85% of commercial Lingzhi products including fruiting body slices, wall-breaking spore, tablets, syrups, and oral liquid are derived from the fruiting body (Zhou et al., 2012). It is more important to enhance the contents of bioactive constituents in the fruiting body of *G. lingzhi*.

High oxygen atmosphere is an effective method in controlling microbiological decay and maintaining the quality of postharvest fruits, vegetables, and mushrooms (Liu and Wang, 2012). Moreover, a high oxygen concentration could also act as an abiotic stress tool to induce signal molecules in fresh fruits, vegetables, and mushrooms, which in turn stimulate the synthesis of secondary metabolites (Liu and Wang, 2012; Van de Velde et al., 2019). Previous researches also showed that ganoderic acid production could be stimulated by a high oxygen concentration or H_2_O_2_ in mycelia of *G. lingzhi* (Zhang et al., 2010; You et al., 2012). However, there is no report on the effect of high oxygen on the bioactive compound production and antioxidant activity of *G. lingzhi* fruiting body. Therefore, the objective of this work was to examine the effects of the application of high oxygen atmosphere on the contents of total phenolics, total flavonoids, and total ganoderic acids of postharvest fruiting body of *G. lingzhi*. Furthermore, the changes of individual ganoderic acid and phenolic contents, the transcriptional levels of key enzyme genes involved in ganoderic acid biosynthesis, free radical scavenging, and DNA protective activities of *G. lingzhi* fruiting body during treatment were also studied.

## Materials and Methods

### Mushrooms and Treatments

Fruiting bodies of *Ganoderma lingzhi* cultivated on beech wood were hand-harvested at a commercially mature stage from a farm located in Zhatian town, Longquan county of Zhejiang Province, China, on August 28, 2018. The fruiting bodies with similar size and maturity, and being free from blemishes and mechanical damage were selected for the investigation.

Two fruiting bodies (about 1 kg) were placed in a 17-L plastic box, and three boxes were used for each treatment. The boxes were placed at 25°C and connected to a continuous flow of air (control), 60% oxygen, and 80% oxygen, respectively. Air was provided by a TP103 air compressor (Ruian Itop Electromechanical Co., Ltd., Ruian, China), and 60 and 80% oxygen (balanced with N_2_ in all high oxygen treatments) was provided by Ouyuan Gas Equipment Co., Ltd. (Wenzhou, China). An oxygen concentration in the boxes was checked regularly with a NGP40-02 oxygen analyzer (Gaopin Science, Ningbo, China) and maintained at ±2% during the treatment by adjusting the flow velocity. Samples were taken every day during the 6 days treatment and stored at −70°C until analysis. For measurements of bioactive compounds and antioxidant and DNA damage protective activities, the samples were ground by a SCIENTZ-48 tissue grinder (Ningbo Xinzhi Biotechnology Co., Ltd., Ningbo, China) at 70 Hz for 90 s and freeze-dried by a LyoQuest-85 freeze dryer (Telstar Technologies, Barcelona, Spain).

### Chemicals

2,2′-Azino-bis(3-ethylbenzothiazoline-6-sulfonic acid) diammonium salt (ABTS), 2,2-diphenyl-1-picrylhydrazyl (DPPH), acetonitrile (high-performance liquid chromatography [HPLC] grade), and the standard syringic acid and vanillic acid were purchased from Sigma-Aldrich (St. Louis, United States). The standard ganoderic acids A, B, C1, and C2 and ganoderenic acid B (≥97%) were purchased from Baoji Chenguang Biotechnology (Baoji, China). The standard protocatechuic acid and gallic acid were purchased from TargetMol (Boston, United States). The standard rutin was purchased from Dr. Ehrenstorfer (Augsburg, Germany). The standard quercetin-3-D-galactoside, coumaric acid, cinnamic acid, quercetin, kaempferol, and hydroxybenzoic acid were purchased from Shanghai YuanYe Biotechnology (Shanghai, China). pBR322 plasmid DNA was purchased from Sangon Biotech. (Shanghai, China). RNA Isolation kit, TransScript All-in-One First-Strand cDNA Synthesis SuperMix kit, and other qPCR reagents were purchased from TransGen Biotech (Beijing, China). Ascorbic acid, pyrogallol, hydrogen peroxide, and all other chemicals and solvents were purchased from Shanghai Chemical Reagent Company (Shanghai, China).

### Phenolic Compound Analysis

One gram of pulverized sample was extracted twice with 30 ml of methanol in a KQ-300VDV ultrasonic cleaner (Kunshan Ultrasonic Instruments, Kunshan, China) at 45 kHz, 50°C, and 210 W for 30 min; and the extract was obtained by centrifugation at 12,000 *g* for 20 min. The total phenolic content was analyzed using the Folin–Ciocalteu method as described by Li et al. (2019) using gallic acid as a standard, and phenolic contents were expressed as grams of gallic acid equivalents per kilogram of dry weight (g GAE kg^–1^). For quantitative analysis of individual phenolic compounds, the extraction was filtered through a 0.45 μm filter, and the contents of individual phenolic compound were determined using an Agilent 1260 Infinity II HPLC equipped with Zorbax SB-C18 column (4.6 × 250 mm, 5 μm, Agilent) and UV/diode array detector (DAD) as described in our previous work (Dong et al., 2019). Calibration curves were generated using the standard solutions of individual phenolic compounds by plotting the peak area versus concentration, and the contents of gallic acid, kaempferol, protocatechuic acid, quercetin, rutin, and syringic acid were quantified by external standard method and based on dry weight, respectively.

### Ganoderic Acid Analysis

The total ganoderic acid content was determined as described previously (Yang et al., 2013), and thymol was used as a standard.

Individual ganoderic acids were extracted and determined using an Agilent 1260 Infinity II HPLC equipped with Zorbax Extend-C18 column (4.6 × 250 mm, 5 μm, Agilent) and UV/DAD as described in our previous work (Dong et al., 2019). Calibration curves were generated using the standard solutions of individual ganoderic acids by plotting the peak area versus concentration, and the contents of ganoderic acids A, B, C1, and C2 and ganoderenic acid B were quantified by external standard method and based on dry weight, respectively.

### Quantitative Real-Time PCR Analysis of Ganoderic Acid Synthesis Gene Expression

Sample (0.1 g) was added to 0.5 ml of TRIzol and ground by a SCIENTZ-48 tissue grinder (Ningbo Xinzhi Biotechnology Co., Ltd., Ningbo, China) at 70 Hz for 60 s. RNA was extracted using an RNA Isolation Kit, treated with RNase-free DNaseI, and then reverse-transcribed to cDNA using a TransScript All-in-One First-Strand cDNA Synthesis SuperMix according to the manufacturer’s protocol. The transcript levels of hydroxymethylglutaryl-CoA synthase (*hmgs*), hydroxymethylglutaryl-CoA reductase (*hmgr*), farnesyl pyrophosphate synthase (*fps*), squalene synthase (*sqs*), and oxidosqualene cyclase (*osc*) were determined by quantitative real-time PCR (qPCR) based on relative method on the Roche LightCycler^®^480 II (Roche Applied Science, Germany). Primer sequences of *hmgs*, *hmgr*, *fps*, *sqs*, and *osc* were described by Ren et al. (2010) and synthesized by Sangon Biotech (Shanghai, China).

PCRs were performed with TransStart Top Green qPCR SuperMix according to the manufacturer’s protocol, and the reaction conditions were described by Xu et al. (2010a). Transcript levels were normalized against the *G. lingzhi* 18S rRNA as an internal control because its expression was stable under the experimental conditions (Xu et al., 2010a). Postquantitative reverse transcriptase (qRT)-PCR calculations analyzing the relative gene expression levels were performed according to the 2^–Δ^ ^Δ^ ^CT^ method. For each gene, the expression of the reference sample was defined as 1.0, and results were expressed as the fold of an mRNA level over the reference sample.

### Measurements of Radical Scavenging Activities

Pulverized sample (0.5 g) was extracted with 15 ml of 80% ethanol in an ultrasonic cleaner at 45 kHz, 50°C, and 210 W for 30 min; and the extract was obtained by centrifugation at 12,000 *g* for 20 min. The scavenging abilities with 2,2′-azino-bis(3-ethylbenzothiazoline-6-sulfonic acid) radical (ABTS^⋅^) and superoxide radical (O_2_^–⋅^) were analyzed as described in our previous work (Wu et al., 2018) and respectively expressed as Trolox equivalents per kilogram of dried *G. lingzhi* (g TE kg^–1^). The analysis of 1,1-diphenyl-2-picrylhydrazyl radical (DPPH^⋅^) and hydroxyl radical (OH^⋅^) scavenging abilities was performed according to our previous work (Wu et al., 2018) with minor modification. For DPPH^⋅^ scavenging measurement, 0.5 ml of properly diluted extract was added to 2.0 ml of DPPH methanol solution (0.2 mmol L^–1^) and 1.5 ml of 80% ethanol. The absorbance was measured at 517 nm after a 30 min reaction at 30°C in the dark. The DPPH radical scavenging ability was calculated and expressed as gallic acid equivalents per kilogram of dried *G. lingzhi* (g GAE kg^–1^). To determine OH^⋅^ scavenging activity, 1.0 ml of properly diluted extract was mixed with 1.0 ml of H_2_O_2_ (6 mmol L^–1^) and 1.0 ml of FeSO_4_ (6 mmol L^–1^), and then 1.0 ml salicylic acid (6 mmol L^–1^) was added after 10 min. The absorbance at 510 nm was measured after a 30 min reaction (37°C) and subsequent centrifugation (4,000 *g*, 10 min). The scavenging ability with O_2_^–⋅^ was calculated and expressed as Trolox equivalents per kilogram of dried *G. lingzhi* (g TE kg^–1^).

### Analysis of DNA Damage Protective Activity

Pulverized sample (0.1 g) was extracted with 3 ml of ethanol in an ultrasonic cleaner at 45 kHz, 50°C, and 210 W for 30 min; and the extract was obtained by centrifugation at 10,000 *g* for 10 min. The hydrogen peroxide-induced DNA damaged assay was done on pBR322 plasmid DNA as described by Gao et al. (2011), with some modifications. Briefly, 10 μl of extract, 2 μl of pBR322 plasmid DNA (125 mg L^–1^), 4 μl of 1 mM FeSO_4_, and 4 μl of 1 mM H_2_O_2_ were incubated at 37°C for 30 min. After incubation, the reaction mixtures were electrophoresed on 1% agarose gel containing GelStain solution (TransGen Biotech) for 45 min at 120 V. After electrophoresis, the DNA in the gel was visualized and photographed using ZF-258 gel imaging system (Jiapeng Science, Shanghai, China). The intensity of DNA band was analyzed by ImageJ software. Quercetin (300 mg L^–1^) and phosphate buffer were used as a positive control and a negative control, respectively. The amount of supercoiled DNA (undamaged DNA) was expressed as a percentage of total amounts of DNA (supercoiled DNA and oxidative damaged DNA) in each gel lane and calculated as described by Xiao et al. (2014).

### Statistical Analysis

All experiments and determinations were performed in triplicate, and data were analyzed by using Statistics Analysis System (SAS) 8.1 version (SAS Institute Inc., United States).

## Results

### Phenolic Compound Contents

**FIGURE 1:**
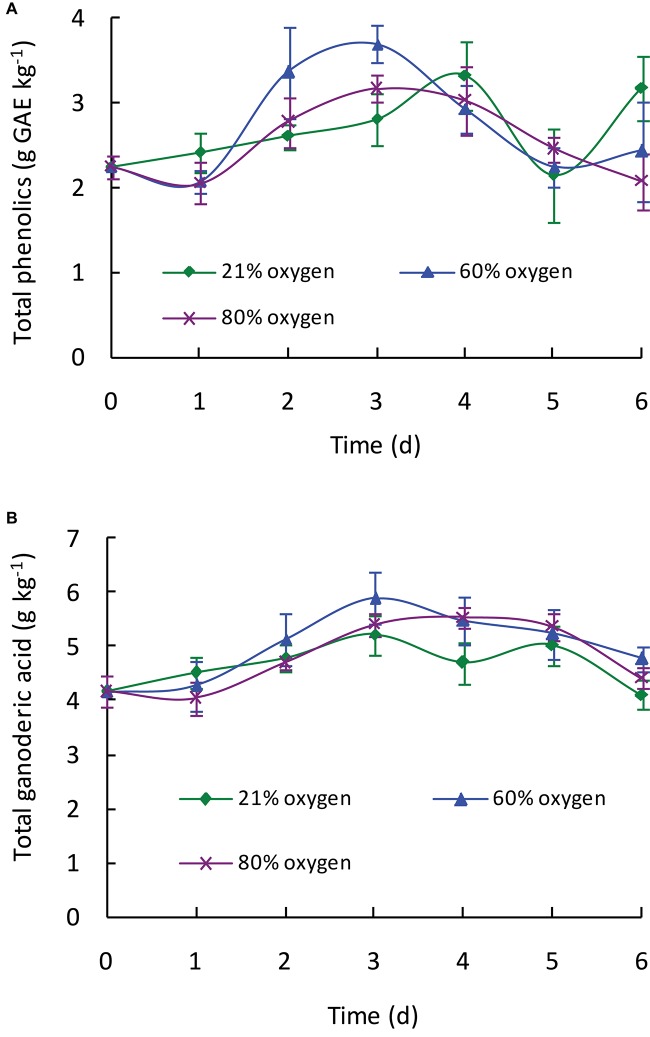
Effect of high oxygen treatment on the total phenolic **(A)** and total ganoderic acid **(B)** contents of *G. lingzhi* fruiting body. Data are expressed as mean ± SD.

As shown in Figure 1A, the total phenolic content in all treated samples exhibited an increasing trend during the treatment of first 3–4 days depending on the different oxygen concentrations. Compared with control, high oxygen treatment could enhance the biosynthesis of phenolic compounds in *Ganoderma lingzhi* fruiting body, and 60% oxygen treatment showed the best effect in which the highest total phenolic content (3.69 g GAE kg^–1^) was determined at day 3, which was 1.31-fold higher than that of control. To investigate the effect of high oxygen treatment on the accumulation of individual phenolic compound, samples were analyzed by HPLC. The HPLC chromatograms of standard phenolic compounds and example of the HPLC profiles of phenolic compounds from *G. lingzhi* are shown in Supplementary Figures S1, S2, respectively. Six phenolic compounds (i.e., gallic acid, protocatechuic acid, syringic acid, rutin, quercetin, and kaempferol) were determined, and the main phenolic compounds were kaempferol and gallic acid. The gallic acid content increased significantly (*p* < 0.05) from initial 444.35 to 653.00 mg kg^–1^ in samples treated with 60% oxygen for 5 days and 717.91 mg kg^–1^ in samples treated with 80% oxygen for 4 days. The kaempferol content increased in the first 2 days and then decreased gradually in samples treated with 80% oxygen. However, the kaempferol biosynthesis was improved significantly in samples treated with 60% oxygen, and the highest content (1,784.08 mg kg^–1^) was determined at day 3, which was 2.12-fold higher than that of control (Table 1).

**TABLE 1:** Effect of high oxygen treatment on the individual phenolic compounds content of *Ganoderma lingzhi* fruiting body (mg kg^–1^).

**Treatment**		**Gallic acid**	**Protocatechuic acid**	**Syringic acid**	**Rutin**	**Quercetin**	**Kaempferol**
Day 0		444.35 ± 1.83	52.46 ± 5.89	9.78 ± 0.69	6.53 ± 0.68	9.99 ± 1.67	623.09 ± 42.59
Day 1	Air	493.18 ± 32.85	61.20 ± 10.32	10.80 ± 1.29	9.95 ± 2.33	9.11 ± 0.14	554.78 ± 26.64
	60%	626.59 ± 2.96	33.97 ± 19.48	11.82 ± 1.60	8.68 ± 1.44	12.82 ± 1.60	930.72 ± 46.79
	80%	542.89 ± 24.12	37.05 ± 14.11	11.74 ± 1.50	13.93 ± 2.76	19.98 ± 0.47	1,317.06 ± 107.90
Day 2	Air	584.83 ± 35.69	34.56 ± 16.85	10.11 ± 1.95	11.01 ± 1.82	11.83 ± 1.37	854.76 ± 45.76
	60%	598.03 ± 18.86	46.74 ± 14.47	10.25 ± 1.94	10.99 ± 0.48	13.13 ± 1.73	965.63 ± 67.30
	80%	523.51 ± 14.53	37.59 ± 8.30	10.12 ± 1.58	12.25 ± 4.71	19.81 ± 3.52	1,304.37 ± 105.60
Day 3	Air	560.42 ± 36.25	64.17 ± 12.26	9.96 ± 1.23	–	17.82 ± 0.44	842.86 ± 61.45
	60%	615.16 ± 40.88	48.07 ± 21.75	10.21 ± 1.29	12.94 ± 0.57	18.13 ± 0.31	1,784.08 ± 199.06
	80%	618.12 ± 33.79	33.40 ± 5.60	10.20 ± 1.47	12.28 ± 2.87	11.61 ± 0.58	977.40 ± 73.81
Day 4	Air	411.10 ± 1.27	128.31 ± 12.49	9.99 ± 2.32	13.58 ± 0.42	12.29 ± 0.11	972.16 ± 98.74
	60%	601.12 ± 32.06	33.13 ± 12.68	11.28 ± 0.11	–	14.87 ± 0.57	940.10 ± 46.08
	80%	717.91 ± 12.67	28.37 ± 18.12	7.46 ± 0.00	14.81 ± 0.44	12.31 ± 0.61	1,140.52 ± 88.10
Day 5	Air	369.29 ± 0.69	108.57 ± 2.00	10.37 ± 0.62	22.05 ± 0.69	13.73 ± 1.34	1,248.98 ± 130.63
	60%	653.00 ± 15.54	112.36 ± 4.29	11.72 ± 0.80	12.43 ± 1.11	11.94 ± 1.14	1,304.93 ± 80.55
	80%	336.93 ± 3.89	98.59 ± 8.02	9.95 ± 0.53	11.41 ± 0.46	5.20 ± 0.07	463.84 ± 26.12
Day 6	Air	484.82 ± 5.08	84.98 ± 8.81	11.63 ± 0.81	20.31 ± 2.80	14.99 ± 1.96	1,309.29 ± 110.28
	60%	256.60 ± 5.31	83.44 ± 0.78	9.76 ± 0.81	9.60 ± 0.49	8.99 ± 0.97	990.23 ± 76.55
	80%	335.18 ± 5.21	73.22 ± 0.77	9.60 ± 0.68	4.50 ± 0.47	7.46 ± 0.14	560.31 ± 22.84

**Data are expressed as mean ± SD.**

### Ganoderic Acid Contents

As shown in Figure 1B, high oxygen treatment could increase the ganoderic acid content of *G. lingzhi* fruiting body, and the suitable operation was treating 3 days with 60% oxygen. Ganoderic acid of 5.89 g kg^–1^ was determined under this condition, which meant a 1.13-fold increase compared with that of control. To investigate the effect of high oxygen treatment on the accumulation of individual ganoderic acid, samples were extracted by chloroform and analyzed by HPLC. The HPLC chromatograms of standard ganoderic acids and example of the HPLC profiles of ganoderic acids from *G. lingzhi* are shown in Supplementary Figures S3, S4, respectively. Ganoderic acids A, B, C1, and C2 were the main ones in the fruiting body of *G. lingzhi*. Although 60% oxygen treatment showed the best effect in most individual ganoderic acids tested, some differences were observed in their contents. The maxima of 1.77 g kg^–1^ of ganoderic acid A and 0.22 g kg^–1^ of ganoderic acid C2 were determined at day 3, while the maximal values (0.39 and 0.25 g kg^–1^, respectively) for ganoderic acid B and ganoderenic acid B were observed at day 4. The contents of ganoderic acids A, B, and C2 and ganoderenic acid B were increased 2. 79-, 2. 32-, 1. 37-, and 2.93-fold by 60% oxygen compared with control, respectively. However, the stimulating effect was not observed in the content of ganoderic acid C1 (Table 2).

**TABLE 2:** Effect of high oxygen treatment on the individual ganoderic acid content of *Ganoderma lingzhi* fruiting body.

**Treatment**	**Individual ganoderic acid (g kg^–1^)**				
	**Ganoderic acid A**	**Ganoderic acid B**	**Ganoderenic acid B**	**Ganoderic acid C1**	**Ganoderic acid C2**
Day 0	0.411 ± 0.054	0.095 ± 0.008	0.061 ± 0.006	0.049 ± 0.005	0.064 ± 0.005
Day 1, air	0.349 ± 0.027	0.065 ± 0.022	0.029 ± 0.009	0.027 ± 0.006	0.041 ± 0.002
60% O_2_	0.384 ± 0.117	0.165 ± 0.032	0.082 ± 0.018	0.060 ± 0.017	0.066 ± 0.013
80% O_2_	0.506 ± 0.099	0.131 ± 0.027	0.084 ± 0.026	0.068 ± 0.012	0.021 ± 0.068
Day 2, air	0.527 ± 0.073	0.212 ± 0.014	0.090 ± 0.008	0.038 ± 0.010	0.093 ± 0.005
60% O_2_	0.601 ± 0.047	0.175 ± 0.024	0.087 ± 0.009	0.046 ± 0.013	0.100 ± 0.007
80% O_2_	0.619 ± 0.032	0.198 ± 0.006	0.100 ± 0.010	0.049 ± 0.007	0.114 ± 0.009
Day 3, air	0.634 ± 0.030	0.251 ± 0.025	0.091 ± 0.005	0.109 ± 0.017	0.138 ± 0.005
60% O_2_	1.774 ± 0.071	0.361 ± 0.031	0.250 ± 0.018	0.086 ± 0.024	0.217 ± 0.007
80% O_2_	1.087 ± 0.062	0.298 ± 0.025	0.196 ± 0.011	0.062 ± 0.008	0.163 ± 0.007
Day 4, air	0.626 ± 0.087	0.168 ± 0.035	0.087 ± 0.016	0.079 ± 0.011	0.085 ± 0.013
60% O_2_	1.218 ± 0.064	0.390 ± 0.089	0.255 ± 0.020	0.046 ± 0.013	0.164 ± 0.019
80% O_2_	0.396 ± 0.032	0.273 ± 0.034	0.080 ± 0.015	0.042 ± 0.005	0.087 ± 0.019
Day 5, air	0.728 ± 0.034	0.210 ± 0.015	0.101 ± 0.013	0.029 ± 0.007	0.104 ± 0.003
60% O_2_	0.594 ± 0.048	0.214 ± 0.036	0.159 ± 0.023	0.062 ± 0.024	0.152 ± 0.007
80% O_2_	0.500 ± 0.056	0.246 ± 0.020	0.089 ± 0.007	0.051 ± 0.019	0.107 ± 0.006
Day 6, air	0.461 ± 0.068	0.138 ± 0.017	0.088 ± 0.006	0.048 ± 0.016	0.101 ± 0.006
60% O_2_	0.869 ± 0.032	0.194 ± 0.010	0.145 ± 0.024	0.071 ± 0.023	0.165 ± 0.007
80% O_2_	0.892 ± 0.083	0.153 ± 0.020	0.144 ± 0.011	0.067 ± 0.014	0.174 ± 0.019

**Data are expressed as mean ± SD.**

### Gene Expressions of Ganoderic Acid Biosynthesis-Related Enzymes

**FIGURE 2:**
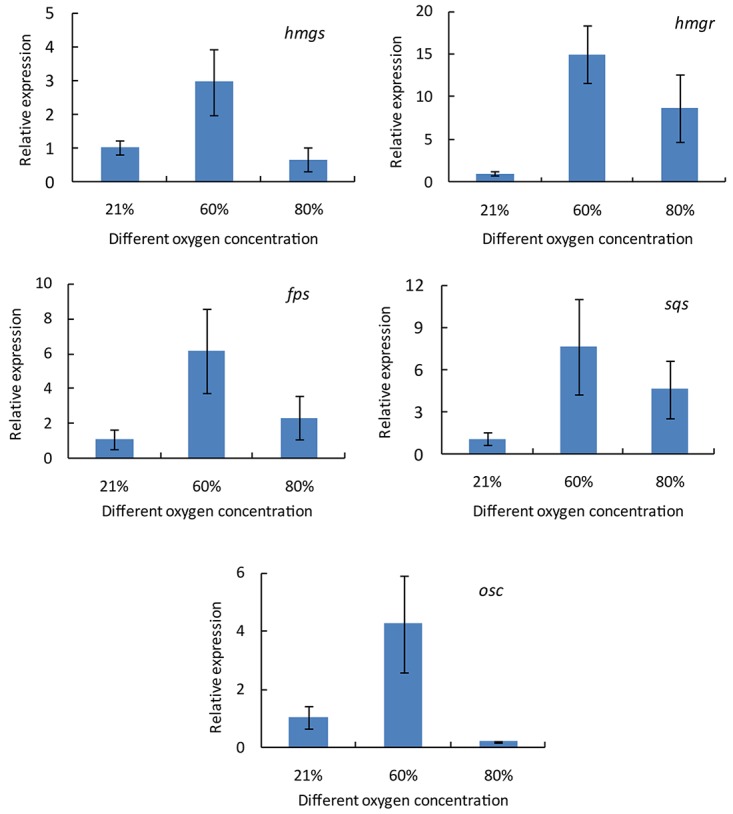
The transcript levels of *hmgs*, *hmgr*, *fps*, *sqs*, and *osc* genes in *Ganoderma lingzhi* fruiting body treated with different oxygen concentrations for 3 days. Data are expressed as mean ± SD.

To explore the molecular mechanism responsible for the increase in the ganoderic acid content, the fruiting bodies of *G. lingzhi* treated with 21 and 60% oxygen concentration were first collected on day 3, and the gene transcription levels of *hmgs*, *hmgr*, *fps*, *sqs*, and *osc* were measured by qRT-PCR. Figure 2 shows that all of these genes were up-regulated in *G. lingzhi* fruiting body treated with 60% oxygen, and the mRNA levels of *hmgs*, *hmgr*, *fps*, *sqs*, and *osc* increased about 2. 95-, 14. 98-, 6. 18-, 7. 61-, and 4.26-fold, respectively, compared with the mRNA level of control (air condition).

**FIGURE 3:**
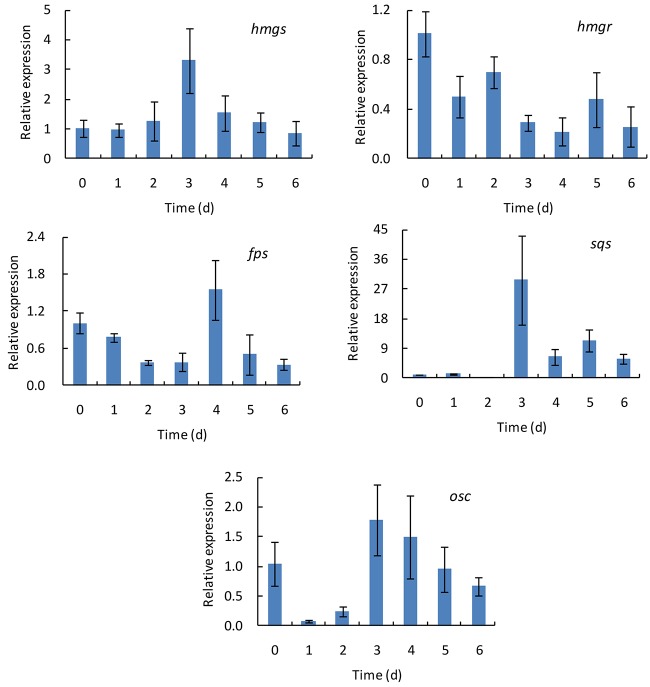
The time course of transcript levels of *hmgs*, *hmgr*, *fps*, *sqs*, and *osc* genes in *Ganoderma lingzhi* fruiting body during the treatment with 60% oxygen for 6 days. Data are expressed as mean ± SD.

To investigate the changes of the transcriptional levels of *hmgs*, *hmgr*, *fps*, *sqs*, and *osc*, *G. lingzhi* fruiting bodies treated with 60% oxygen were sampled at 1 day interval during the treatment of 6 days, while those at day 0 were taken as controls. As shown in Figure 3, the transcript levels of these genes varied as the treating time changed, and the time course of these genes in response to 60% oxygen treatment varied from gene to gene. *hmgs* expression increased gradually in the first 3 days and reached 3.31-fold higher than did that of the control and thereafter decreased. In comparison, no increasing expression level was determined in *hmgr*, and the maximal expression levels of *fps* and *osc* were observed at days 4 and 3, respectively, which only increased 1.55- and 1.79-fold compared with the expression level of control. A much higher expression level was determined in *sqs*, compared with the control, in which a transcriptional level increased 29.79-fold on day 3.

### Free Radical Scavenging Activity

**FIGURE 4:**
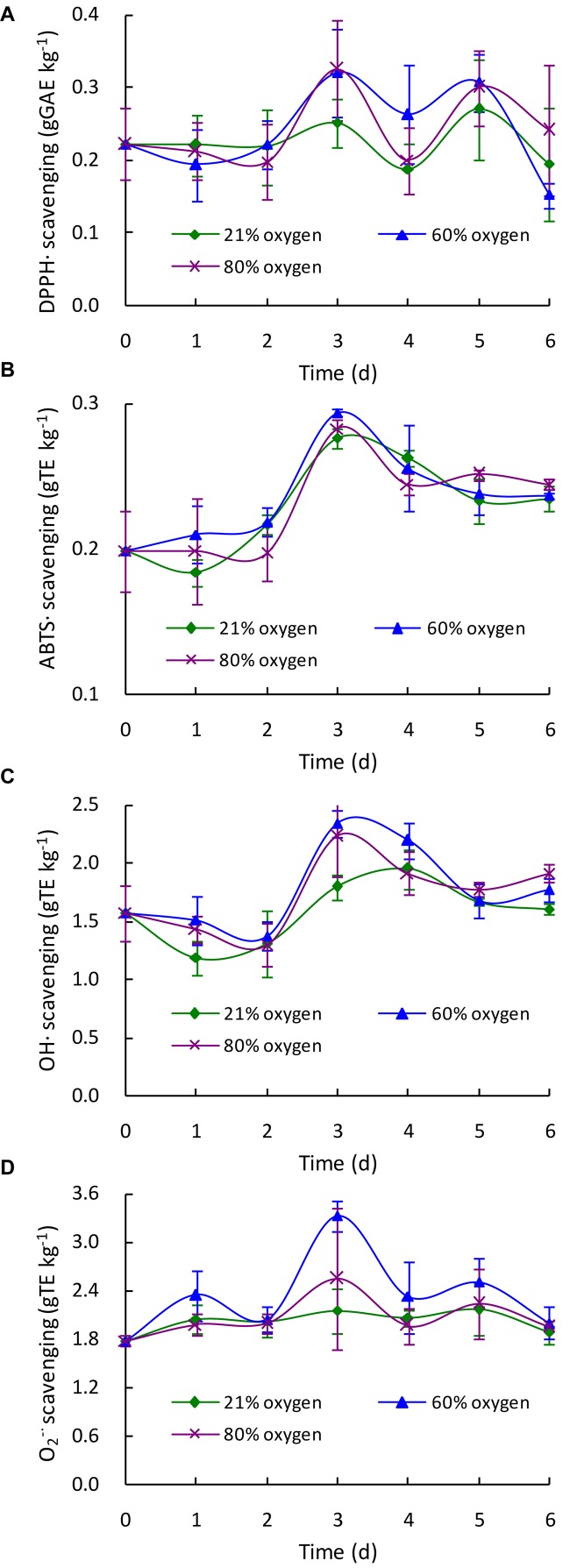
Effect of high oxygen treatment on the DPPH radical **(A)**, ABTS radical **(B)**, hydroxyl radical **(C)**, and superoxide radical **(D)** scavenging activities of *Ganoderma lingzhi* fruiting body. Data are expressed as mean ± SD.

To evaluate the effects of different high oxygen treatments on the free radical scavenging properties of *G. lingzhi*, the DPPH radical (DPPH^⋅^), ABTS radical (ABTS^⋅^), hydroxyl radical (OH^⋅^), and superoxide radical (O_2_^–⋅^) were assayed in the present study. As shown in Figure 4A, the DPPH^⋅^ scavenging activity was markedly increased from 0.22 g GAE kg^–1^ at day 0 to about 0.32 g GAE kg^–1^ at day 3 by 60 or 80% oxygen, suggesting a 1.28-fold increase higher than that of control (0.25 g GAE kg^–1^). The ABTS^⋅^ scavenging ability was gradually increased by 60 or 80% oxygen treatment, and 0.29 g TE kg^–1^ was determined in 60% oxygen treatment group at day 3, which was significantly (*p* < 0.05) higher than that of control. However, no significant difference in ABTS^⋅^ scavenging ability was observed between 80% oxygen group and control (Figure 4B). The highest OH^⋅^ and O_2_^–⋅^ scavenging activities were also determined in 60% oxygen treatment group at day 3, which were 2.34 g TE kg^–1^ for OH^⋅^ scavenging activity and 3.32 g TE kg^–1^ for O_2_^–⋅^ scavenging activity, representing 1.30- and 1.54-fold higher than that of control, respectively (Figures 4C,D).

### DNA Damage Protective Activity

**FIGURE 5:**
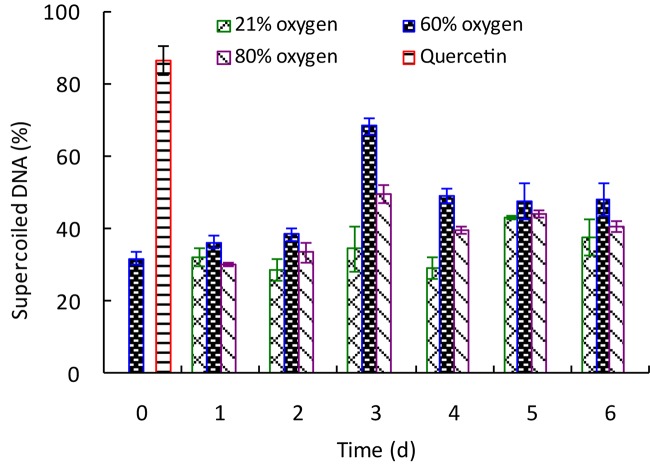
Effect of high oxygen treatment on the DNA damage protective activity of *Ganoderma lingzhi* fruiting body. Percent of supercoiled DNA. Data are expressed as mean ± SD.

To determine the effect of high oxygen treatment on the DNA damage protective property of *G. lingzhi*, samples were extracted and tested for their protective effects on pBR322 plasmid DNA damage caused by hydroxyl radicals generated from Fenton’s reaction. As shown in Supplementary Figure S5, the plasmid DNA was mainly of the supercoiled form in the absence of Fenton’s reagent (line 1) and converted into nicked form by OH^⋅^ generated from iron-mediated decomposition of H_2_O_2_ (line 2). Quercetin (postcontrol, line 3) and *G. lingzhi* extracts showed varying degrees of protection for supercoiled DNA. The percentage intensity of the supercoiled DNA and nicked DNA forms in each reaction is shown in Figure 5. The supercoiled DNA percentage increased gradually to the maxima of 68.74% by 60% oxygen and 49.75% by 80% oxygen at day 3, which was 2.15- and 1.56-fold higher than that of control, respectively, suggesting that high oxygen treatment could enhance the DNA damage protection activity of *G. lingzhi* fruiting body.

## Discussion

*Ganoderma lingzhi* contains a variety of phenolic compounds including *p*-hydroxybenzoic acid, *p*-coumaric acid, cinnamic acid, gallic acid, quercetin, and rutin, which are related to the antioxidant, antimicrobial, and anti-inflammatory activities of *G. lingzhi* (Obodai et al., 2017; Veljović et al., 2017; Saltarelli et al., 2019). Gallic acid, protocatechuic acid, syringic acid, rutin, quercetin, and kaempferol were determined in this work, and the total contents in the control sample (under 21% oxygen concentration) ranged from 1,146.20 to 1,926.02 mg kg^–1^ during the treatment (Table 1), which are much higher than those of the fruiting body of *G. lingzhi* procured from M/s Aryan Enterprises, India (4.13 mg GAE kg^–1^; Mishra et al., 2018), but lower than those provided by BioReishi, Portugal (4.6 g GAE kg^–1^, Oludemi et al., 2018), owing to the different strain and growth conditions.

Plants and mushrooms can produce some stress response and induce the production of reactive oxygen species under biotic and abiotic stress conditions (Sudheer et al., 2016; Zhao et al., 2018), which act as signal molecules to induce the synthesis of antioxidants and other secondary metabolites by regulating signal transduction pathways (Apel and Hirt, 2004). A high oxygen concentration could act as an abiotic stress to induce the biosynthesis of antioxidant enzymes and secondary metabolites in some fruit and mushrooms such as blueberry (Zheng et al., 2003), strawberry (Van de Velde et al., 2019), and *Agaricus bisporus* (Liu and Wang, 2012). Similarly, a high oxygen concentration could also enhance the biosynthesis of phenolic compounds in *G. lingzhi* fruiting body. The optimal condition is treated with 60% oxygen for 3 days, and 3.69 g GAE kg^–1^ of the total phenolic content was determined (Figure 1A).

Ganoderic acids are one of the main bioactive compounds that exist in *G. lingzhi*, and an oxygen level in the atmosphere is a vital factor in the mycelial growth, sporulation, and ganoderic acid biosynthesis. Previous research showed that ganoderic acid synthesis in mycelia of *G. lingzhi* can be stimulated by a high oxygen concentration or H_2_O_2_ (Zhang et al., 2010; You et al., 2012). The mycelia of *G. lingzhi* CCGMC 5.616 grown in 80% oxygen contained ganoderic acid at a concentration of 47 g kg^–1^, which was 30% higher than that in the air condition (Zhang et al., 2010). The ganoderic acid content in the fruiting bodies of *G. lingzhi* treated with 5 ml L^–1^ of ozone fumigation for 48 h reached a maximum of 4.44 g kg^–1^, which was a twofold increase compared with that of control (Sudheer et al., 2016). In accordance with the results of Nakagawa et al. (2018), ganoderic acids A, B, C1, and C2 were determined to be the main ones in this work. High oxygen treatment could also enhance the contents of ganoderic acids. Similar with phenolic compound, 60% oxygen treatment showed the best effect in most individual ganoderic acids tested.

Ganoderic acids of *G. lingzhi* are synthesized via mevalonate-isoprene pathway; and hydroxymethylglutaryl-CoA synthase, hydroxymethylglutaryl-CoA reductase, farnesyl pyrophosphate synthase, squalene synthase, and oxidosqualene cyclase are the key enzymes in this pathway (Ren et al., 2010). qRT-PCR analysis showed that the gene transcription levels of *hmgs*, *hmgr*, *fps*, *sqs*, and *osc* were up-regulated in *G. lingzhi* fruiting body treated with 60% oxygen (Figure 2). A 29.79-fold higher transcriptional level was determined in *sqs*, compared with the control, on day 3 (Figure 3). This is similar with the results of Zhang et al. (2010) who demonstrated that 80% oxygen could induce the expressions of genes related to ganoderic acid biosynthesis in the mycelia of *G. lingzhi* CCGMC 5.616. *sqs* is an important gene in the ganoderic acid biosynthesis pathway (Ren et al., 2010), and overexpression of *sqs* could significantly enhance the production of ganoderic acids (Zhou et al., 2014). An oxygen concentration of 60% in the gaseous phase increased the mRNA level of *G. lingzhi* fruiting body, and it was concluded that the high expression levels of *sqs* gene, compared with the ganoderic acid production, were correlated with a high content of ganoderic acid.

Free radicals are involved in the lethal peroxidation damage to bio-molecules and thought to play an important role in the progression of many chronic diseases such as cancer, neurodegenerative disorders, and cardiovascular diseases (Abbas et al., 2014). Numerous researches showed that *G. lingzhi* could scavenge various free radicals and reactive oxygen species due to its rich phenolic, triterpenoid, and polysaccharide compounds (Wang et al., 2013; Mishra et al., 2018; Saltarelli et al., 2019). In this work, the changes of DPPH radical (DPPH^⋅^), ABTS radical (ABTS^⋅^), hydroxyl radical (OH^⋅^), and superoxide radical (O_2_^–⋅^) scavenging activities in *G. lingzhi* were evaluated during the treatment, and enhancement of free radical scavenging activity was observed in *G. lingzhi* fruiting body treated with 60% oxygen for 3 days (Figure 4), in which the contents of phenolic compounds and ganoderic acids are increased (Figures 1, 2). During the treatment with 60% oxygen, the DPPH^⋅^ scavenging ability of *G. lingzhi* is correlated with the contents of gallic acid (*R* = 0.704) and quercetin (*R* = 0.716), and the contents of kaempferol decide the ABTS^⋅^ and O_2_^–⋅^ scavenging activities, in which the correlation coefficient is 0.867 and 0.946, respectively. Quercetin also plays an important role in ABTS^⋅^ and O_2_^–⋅^ scavenging activities (*R* = 0.760 and 0.853, respectively). In addition to phenolic compounds, ganoderic acids also have strong free radical scavenging activity (Smina et al., 2011). High correlation coefficients were observed between ABTS^⋅^, O_2_^–⋅^, and OH^⋅^ scavenging activities and the contents of ganoderic acids A, B, and C2 and ganoderenic acid B in *G. lingzhi* fruiting body treated with 60% oxygen (Table 3).

**TABLE 3:** Correlation coefficients (*R*) between radical scavenging activity (DPPH^⋅^, ABTS^⋅^, O_2_^–⋅^, and OH^⋅^ scavenging activities) and the contents of phenolic compounds and ganoderic acids in *Ganoderma lingzhi* fruiting body during treatment with 60% oxygen.

**Radical scavenging**	**DPPH^⋅^**	**ABTS^⋅^**	**O_2_^–⋅^**	**OH^⋅^**
**activity**	**scavenging**	**scavenging**	**scavenging**	**scavenging**
Gallic acid	0.704	0.217	0.519	0.104
Kaempferol	0.698	0.867	0.946	0.622
Protocatechuic acid	0.142	0.013	−0.031	−0.152
Syringic acid	0.303	−0.016	0.238	−0.031
Rutin	0.194	0.145	0.384	−0.219
Quercetin	0.716	0.760	0.853	0.671
Ganoderic acid A	0.533	0.964	0.774	0.932
Ganoderic acid B	0.579	0.889	0.702	0.901
Ganoderenic acid B	0.604	0.926	0.707	0.942
Ganoderic acid C1	0.260	0.668	0.735	0.512
Ganoderic acid C2	0.522	0.948	0.712	0.835

DNA damage might ultimately lead to carcinogenesis, mutagenesis, and cytotoxicity (Xiao et al., 2014), and accumulating reports show the protective effect of plant-derived extracts or compounds against oxidative DNA damage (Xiao et al., 2014; Limmongkon et al., 2019). *In vitro* and *in vivo* experiments demonstrate that *G. lingzhi* has significant effectiveness in protecting the DNA damage by radical scavenging, interaction with apurinic/apyrimidinic endonucleases, or restoration of enzymatic antioxidant activity due to the ample source of triterpenes, polyphenols, and other active ingredients (Smina et al., 2015; Kumari et al., 2016; Saltarelli et al., 2019). Similar with free radical scavenging activity, the DNA damage protective property of *G. lingzhi* could also be improved by high oxygen treatment (Figure 5), and the protective activity against the plasmid DNA damage was found to have a significant correlation (*R*^2^ = 0.855) with the hydroxyl radical scavenging capacity in *G. lingzhi* fruiting body treated with 60% oxygen (Figure 4).

## Conclusion

The bioactive compounds of *Ganoderma lingzhi* are widely used as nutraceuticals, and various methods have been employed to enhance their biosynthesis. From this study, high oxygen was beneficial to enhance the contents of phenolic compound and ganoderic acid. The optimal operation was treating *G. lingzhi* fruiting body with 60% oxygen for 3 days, and the transcriptional levels of ganoderic acid synthesis-related enzymes were induced under this condition. The free radical scavenging with DPPH^⋅^, ABTS^⋅^, OH^⋅^, and O_2_^–^^⋅^ and DNA damage protective activity were also increased at different degrees by 60% oxygen. Based on these results, it is possible to employ a high oxygen concentration to induce the bioactive compound synthesis, resulting in enhanced bioactivity and quality of *G. lingzhi* fruiting body.

## Data Availability Statement

The raw data supporting the conclusions of this manuscript will be made available by the authors, without undue reservation, to any qualified researcher.

## Author Contributions

GL and HY designed the study. QD, YL, ZZ, and HZ performed the experiments and analyzed the data. HY wrote the manuscript.

## Conflict of Interest

The authors declare that the research was conducted in the absence of any commercial or financial relationships that could be construed as a potential conflict of interest.
