# Supplementary material for: High Oxygen Treatments Enhance the Contents of Phenolic Compound and Ganoderic Acid, and the Antioxidant and DNA Damage Protective Activities of Ganoderma lingzhi Fruiting Body
Source: Front Microbiol. 2019 Oct 18;10:2363. doi: 10.3389/fmicb.2019.02363 (PMC6813255; doi:10.3389/fmicb.2019.02363)
Supplement: Supplementary file 1 [file Table_1.DOCX]

Supplementary Figures


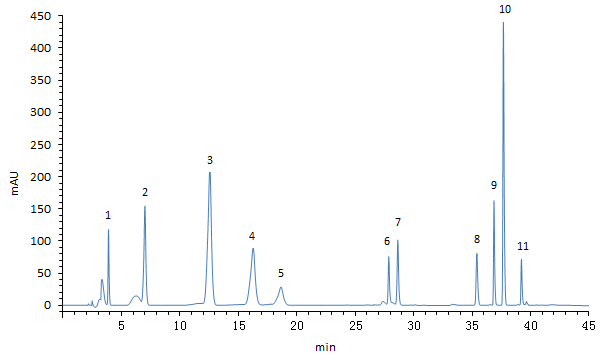


Figure S1. The HPLC chromatograms of standard phenolic compounds.1, gallic acid; 2, protocatechuic acid; 3, hydroxybenzoic acid; 4, vanillic acid; 5, syringic acid; 6, rutin; 7, quercetin-3-D-galactoside; 8, coumaric acid; 9, quercetin; 10, cinnamic acid; 11, kaempferol


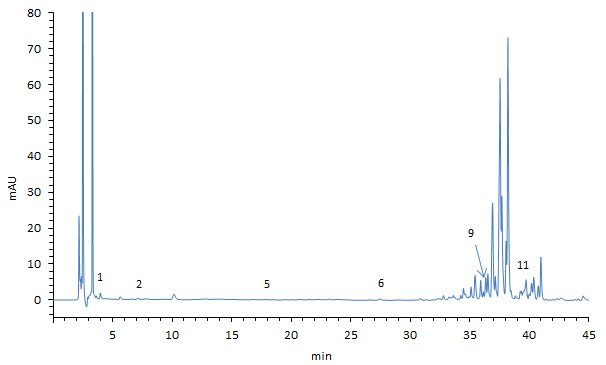


Figure S2. Example of the HPLC profiles of phenolic compounds from *G. lingzhi*. 1, gallic acid; 2, protocatechuic acid; 3, hydroxybenzoic acid; 4, vanillic acid; 5, syringic acid; 6, rutin; 7, quercetin-3-D-galactoside; 8, coumaric acid; 9, quercetin; 10, cinnamic acid; 11, kaempferol


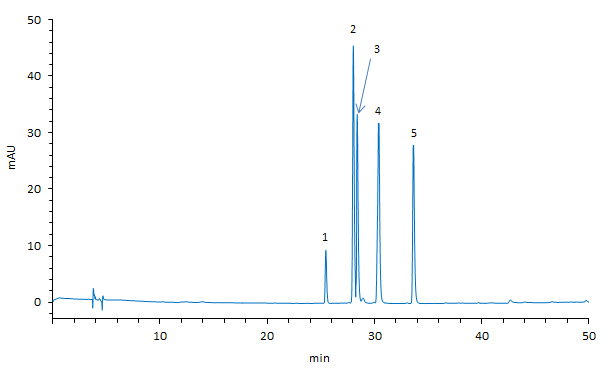


Figure S3. The HPLC chromatograms of standard ganoderic acids.1, ganoderic acid C2; 2, ganoderenic acid B; 3, ganoderic acid B; 4, ganoderic acid A; 5, ganoderic acid C1.


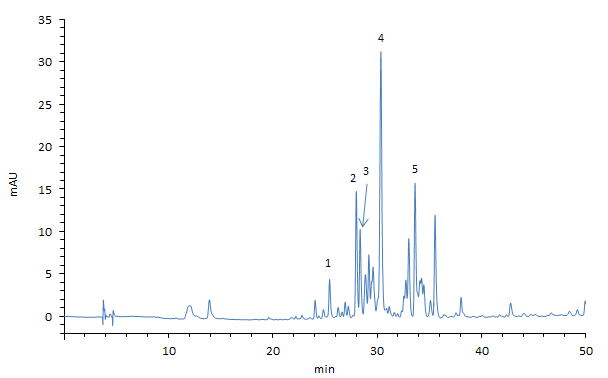


Figure S4. Example of the HPLC profile ofganoderic acids from *G. lingzhi* fruiting body.1, ganoderic acid C2; 2, ganoderenic acid B; 3, ganoderic acid B; 4, ganoderic acid A; 5, ganoderic acid C1.


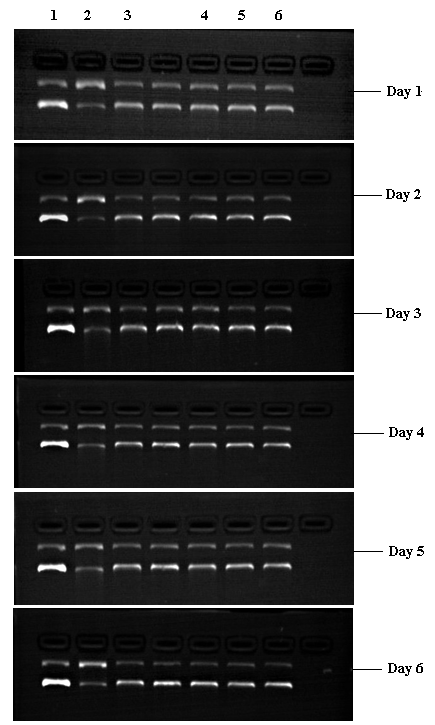


Figure S5. Effect of high oxygen treatment on the DNA damage protective activity of *G. lucidum* fruiting body. gel electrophoresis. Line 1, native pBR322 plasmid DNA + PBS; Line 2, DNA + Fenton’s reagent; Line 3, DNA + Fenton's reagent + Quercetin; Lines 4-6, DNA + Fenton's reagent + Extracts of samples treated by 21, 60, and 80 % oxygen.
